# Plasma tumor necrosis factor-α is associated with sarcopenia in elderly individuals residing in agricultural and pastoral areas of Xinjiang, China

**DOI:** 10.3389/fmed.2022.788178

**Published:** 2022-09-08

**Authors:** Aishanjiang Wumaer, Zhuoya Maimaitiwusiman, Wenwen Xiao, Saiyare Xuekelati, Jinling Liu, Tajiguli Musha, Hongmei Wang

**Affiliations:** Second Department of the Cadre Health Care Center, People’s Hospital of Xinjiang Uygur Autonomous Region, Urumqi, China

**Keywords:** elderly population, inflammatory cytokines, plasma tumor necrosis factor-α, sarcopenia, Xinjiang

## Abstract

**Background:**

Inflammatory reactions play a significant role in the occurrence and development of sarcopenia. Determining the association between specific cytokines and sarcopenia may reveal the disease’s pathophysiological mechanism(s). Accordingly, the present study aimed to investigate the association between sarcopenia and inflammatory cytokines among the elderly natural population in agricultural and pastoral areas of Xinjiang.

**Methods:**

We conducted a cross-sectional epidemiological survey of the community-dwelling older people using a multi-stage random sampling method in Mulei County in northern Xinjiang and Luopu County in southern Xinjiang from September 2017 to May 2018. Of the 2,100 participants, the statistical analyses included 1,838 participants with complete data. Comparisons of living habits, disease status, biochemical indexes, and levels of interleukin (IL)-4, IL-6, IL-8, IL-10, and tumor necrosis factor (TNF)-α in sarcopenia and non-sarcopenia participants were made in this study.

**Results:**

Our study revealed no significant differences (i.e., *P* > 0.05) in sex, age, ethnicity, smoking and drinking habits, serum renal function, total cholesterol, and diabetes in the elderly between the sarcopenia and non-sarcopenia groups in Xinjiang. However, triglyceride levels (*P* = 0.004), hypertension (*P* = 0.019), and abdominal obesity (*P* < 0.001) in the sarcopenia group were significantly higher than those in the non-sarcopenia group. Moreover, the levels of IL-10 (*P* < 0.001), IL-4 (*P* < 0.001), and TNF-α (*P* < 0.001) in the sarcopenia group were higher than those in the non-sarcopenia group after adjusting for sex, age, hypertension, blood lipid concentration, and obesity. Furthermore, after adjusting for sex, age, hypertension, obesity, and IL-10, IL-4, and IL-6 levels, an increased TNF-α level was also significantly associated with sarcopenia.

**Conclusion:**

The results of the present study suggest that an increased plasma level of TNF-α is significantly associated with sarcopenia among elderly individuals residing in Xinjiang’s agricultural and pastoral areas. Further study is still needed to determine the physiological role of “immune aging” in the pathogenesis of sarcopenia.

## Introduction

China has gradually witnessed the emergence of an aging population since 2000; accordingly, the incidence of various chronic diseases has been increasing (1). Sarcopenia has become a significant public health problem. Sarcopenia is a systemic condition involving muscle mass reduction and/or decline in muscle strength and function. It seriously endangers the health of elderly individuals and significantly reduces their quality of life. Individuals with sarcopenia have more difficulty with daily activities and are at a higher risk of infection, fall(s), disability, and/or death (2).

Sarcopenia is characterized by lower levels of exercise tolerance and decreased neuromuscular function. Its etiology includes the following: age-related hormonal changes, increased levels of pro-inflammatory cytokines, myocyte apoptosis, genetic factors, and low nutrient intake (3, 4). Previous studies have reported that the prevalence of sarcopenia in elderly individuals is approximately 10–20% and increases with age, with a prevalence of 5–13% in those 60–70 years of age and 50% in the elderly, i.e., in people > 80 years of age (5–7). In addition, due to the close relationship between sarcopenia and common diseases, the difficulty of treatment increases for patients with comorbid diseases, the period of hospitalization is prolonged, and the burden of social care and medical expenses increases significantly (8, 9).

Therefore, discovering the potential pathophysiological mechanisms of sarcopenia has become increasingly vital, gaining consensus. Recently, some studies have reported that the occurrence and development of sarcopenia and the subsequent deterioration of individuals with this disease are all accompanied by different degrees of inflammatory reactions (10–12). More specifically, a variety of cytokines, including tumor necrosis factor (TNF)-α, interleukin (IL)-1, IL-2, IL-4, IL-6, IL-8, and IL-10 play a role in the development of sarcopenia (13, 14). However, to our knowledge, the current literature on the relationship between sarcopenia and inflammatory cytokines remains unclear. Therefore, the present study explores the relationship between sarcopenia and IL-4, IL-6, IL-10, and TNF-α in the elderly population of agricultural and pastoral areas of Xinjiang, China, and is expected to lay a preliminary foundation for understanding its underlying mechanism.

## Materials and methods

### Study participants

The present investigation was a case-control study based on a cross-sectional epidemiological survey of sarcopenia in agricultural and pastoral areas of Xinjiang. The epidemiological survey was performed from September 2017 to May 2018 using a multi-stage random sampling method. In the first stage, two counties, Mulei County in Northern Xinjiang and Luopu County in southern Xinjiang, were selected; in the second stage, six towns were randomly selected from each county; in the third stage, five villages were randomly selected from each township; and, in the fourth stage, 35 elderly individuals were randomly selected using random number software from each village, which administrative village with household registration base books were provided by the relevant departments, followed by numbering the households in a particular order. A total of 2,100 subjects were included, of whom 1,838 completed the survey, corresponding to a response rate of 87.52%.

**FIGURE 1:**
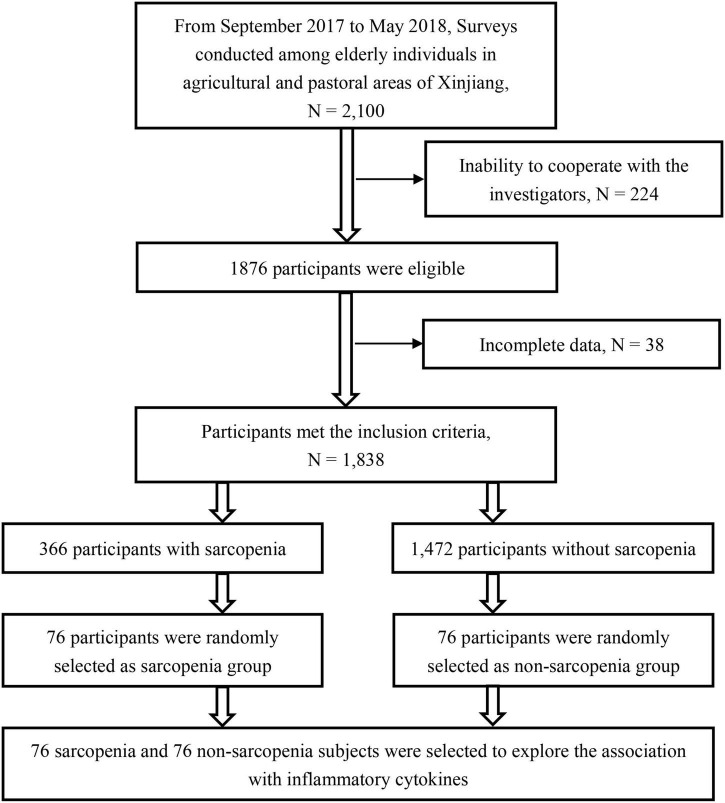
Selection procedure of the study participants.

A total of 152 elderly individuals ≥ 60 years of age were randomly selected from a database established based on the above epidemiological survey using computer-generated balanced block randomization and randomized 1:1 to two groups: sarcopenia (*n* = 76) and non-sarcopenia (*n* = 76). All subjects provided informed consent to participate. The present study was reviewed and approved by the Medical Ethics Committee of Xinjiang Uygur Autonomous Region People’s Hospital. The selection procedures of our study are as follows (Figure 1).

### Inclusion/exclusion criteria

Individuals fulfilling the following criteria were included in the study: age ≥ 60 years; permanent residents with local household registration; able to walk independently without using assistive devices; and informed consent. Individuals with severe cognitive impairment, mental illness, a history of major organ failure (such as respiratory failure, heart failure, renal failure, and liver failure), recent surgical history, and consumptive disease(s) such as malignant tumor and/or tuberculosis were excluded.

### Questionnaire survey

All subjects provided informed consent to participate and completed the questionnaire under the guidance of investigators. The questionnaire addressed age, sex, disease, medication, family and menstrual histories, regular exercise patterns and amount of exercise, fall(s), alcohol consumption, dietary habits, and smoking history.

### Physical examination

The following parameters were measured by trained and qualified professionals in accordance with standard methods: blood pressure, weight, height, waist circumference, hip circumference, and body mass index (BMI). BMI was calculated as weight (kg) divided by height (m) squared (i.e., kg/m^2^). Blood pressure levels were measured using an automatic blood pressure monitor. Readings were obtained after 5 min of seated rest. Three blood pressure measurements were obtained at 30-s intervals. The mean of all available measurements was used to define the systolic and diastolic blood pressure levels. In addition, the following indicators were measured.

### Skeletal muscle strength

The grip strength test was performed using a handheld dynamometer (Jamar, Duluth, MN, United States), and the results reflected skeletal muscle strength. Before the test, the grip distance was adjusted to within the proper range. During the test, the subjects were seated, with the upper arm and forearm angled at 90^°^, and the test arm was slightly extended but not > 30°. The instrument was gripped with maximum force using the right and left hands twice. Maximum values were recorded, and in cases where the data difference between the two sides was small, statistical analysis was conducted with data gathered for the right hand.

### Skeletal muscle quality

A body composition analyzer (InBody720, InBody Co., Ltd., Cerritos, CA, United States) was used to assess skeletal muscle quality in the subjects’ limbs. Subjects fasted for 2 h before measurement. During the test, the subjects were required to empty their bladders, rest for 1 h, and then stand still for 5 min. Subsequently, they removed their shoes and socks, and a small amount of alcohol was applied to the soles of their feet and fingers. Subjects stood on the foot electrodes of the test platform and were held by electrodes placed in the palms of both hands. Their arms were separated from the trunk by approximately 30^°^, and their bodies remained still during the test.

### Skeletal muscle activity/function

Skeletal muscle activity was ascertained by measuring the maximum walking speed of the subjects. The colored tape was used to mark a 16-m straight line, with markings at the starting point, 3.00 m, 13.00 m, and the end. When subjects reached the 3.00-m point, the timing was started, and when they reached 13.00 m, the timing was ended. Three tests were conducted, and the fastest was included in the statistical analysis.

### Collection of blood samples and laboratory testing

Blood was collected from all subjects using disposable blood collection equipment to extract 10 ml of sample (fasting at least 10 h). Sampling was performed in the morning, and all samples were centrifuged immediately to separate plasma (serum) and blood cells. Plasma and blood cells were stored at –80°C until use.

A biochemical analysis system (Hitachi 7600 Clinical Series Analyzer, Hitachi, Tokyo, Japan) was used to assess blood lipid and glucose levels and other biochemical indicators, including fasting blood glucose (FBG), serum creatinine and urea nitrogen, total cholesterol (TC), total triglyceride (TG), high-density lipoprotein cholesterol, and low-density lipoprotein cholesterol.

### Inflammatory cytokines

The levels of IL-6, IL-4, IL-10, and TNF-α were determined using commercially available ELISA kits [MULTISCIENCES (LIANKE) BIOTECH, CO., LTD, Hangzhou, China] as per the manufacturer’s instructions.

### Related diagnostic criteria

#### Sarcopenia

According to the diagnostic criteria for sarcopenia from the 2014 Asian Sarcopenia Working Group (AWG) (15), subjects meeting any two of the following criteria were considered sarcopenia patients: (i) decreased skeletal muscle mass (male < 7.0 kg/m^2^, female < 5.7 kg/m^2^); (ii) decreased grip strength (male < 26 kg, female < 18 kg); and (iii) decreased walking speed (<0.8 m/s).

#### Hypertension

Hypertension was diagnosed based on the Chinese Guidelines for the Prevention and Treatment of Hypertension (revised in 2018). For patients not using antihypertensive drugs, consultation room blood pressure was measured three times on different days, with systolic blood pressure ≥ 140 mmHg and diastolic blood pressure ≥ 90 mmHg. Patients diagnosed with hypertension and taking antihypertensive drugs were also monitored.

#### Type II diabetes

According to the Chinese Guidelines for the Prevention and Treatment of Type II Diabetes Mellitus, 2017 Edition, patients with (i) typical diabetic symptoms (polydipsia, polyuria, overeating, unexplained weight loss) and random blood glucose ≥ 11.1 mmol/L; (ii) FBG ≥ 7.0 mmol/L; (iii) blood glucose at 2 h after glucose loading > 11.1 mmol/L; (iv) and patients diagnosed with type 2 diabetes and taking hypoglycemic drugs were monitored. Patients without typical symptoms of diabetes were re-examined on another day.

#### Abdominal obesity

According to the National Cholesterol Education Program (ATP-III), abdominal obesity was defined as waist circumference ≥ 102 cm (40 inches) for men and 88 cm (35 inches) for women.

### Statistical analysis

SPSS version 17.0 (SPSS Inc., Chicago, IL, United States) was adopted for data processing. Measurement data are expressed as mean ± standard deviation, and count data are expressed as the number of cases or percentages. Comparison of clinical phenotypic measurement data between groups was performed using a *t*-test (between two groups), and that for the classification data was performed using a chi-squared test. Covariance analysis was used to explore the correlation between sarcopenia and inflammatory cytokines in the control and case groups after adjusting for sex, hypertension, blood lipids, and obesity. Binary logistics analysis was used to explore independent factors for sarcopenia in this population. To investigate the differences of four serum inflammatory cytokines between the sarcopenia group and the non-sarcopenia group, a partial least squares-discriminant analysis (PLS-DA) was initially performed using MetaboAnalyst (version 5.0).^1^ Data of serum inflammatory cytokines were abundances normalized by median, log-transformed, and auto-scaled before PLS-DA. Then, a variable selection approach named variable importance in projection (VIP) was used to detect the essential inflammatory cytokines associated with case patients. In addition, the classification models were performed. Statistical significance was identified using an area under the receiver operating characteristic curve (AUROC). The discriminant Q2 under the null hypothesis was calculated through a 1,000-repetition permutation test. Differences with *P* < 0.05 were statistically significant.

## Results

### Clinical characteristics

There were no statistically significant differences in sex, age, ethnicity, smoking, drinking, serum renal function, TC, and diabetes between the sarcopenia and non-sarcopenia groups (i.e., *P* > 0.05); however, there were significant differences in BMI, FBG, hypertension, TG levels, and abdominal obesity (all *P* < 0.05) (Table 1).

**TABLE 1:** Clinical data analysis of subjects.

Variables	Sarcopenia (*N* = 76)	Non-sarcopenia (*N* = 76)	*t(χ^2^)*	*P*
Age (years)	71.0 ± 4.6	70.0 ± 3.8	1.443	0.151
Gender (male,%)	42.1	43.4	0.027	1.000
BMI (kg/m^2^)	26.5 ± 3.0	20.9 ± 2.8	–11.839	< 0.001
Nationality (Han/Uygur)	38/38	38/38	0.000	1.000
Diabetes (yes, %)	11.8	25.0	4.378	0.058
Hypertension (yes, %)	51.3	71.1	6.233	0.019
Coronary heart disease (yes, %)	17.1	10.5	1.381	0.240
Stoke (yes, %)	6.6	5.3	0.118	1.000
Smoking (yes, %)	14.5	14.5	0.000	1.000
Drinking (yes, %)	10.5	11.8	0.066	1.000
Abdominal obesity (yes, %)	51.3	88.2	24.440	< 0.001
Serum creatinine (mg/dl)	62.7 ± 17.0	65.4 ± 17.4	0.941	0.348
Serum urea nitrogen (mmol/L)	5.8 ± 1.5	6.2 ± 1.6	1.461	0.146
Fasting blood glucose (mmol/L)	6.5 ± 2.7	5.5 ± 1.2	–2.779	0.006
Total cholesterol (mmol/L)	4.4 ± 0.9	4.6 ± 1.0	1.218	0.225
Triglyceride (mmol/L)	1.2 ± 0.6	1.7 ± 1.4	2.920	0.004
High-density lipoprotein cholesterol (mmol/L)	1.5 ± 1.3	1.5 ± 0.7	–0.165	0.869
Hemoglobin (g/L)	143.1 ± 27.4	141.6 ± 20.0	–0.288	0.774
Albumin (g/L)	43.4 ± 5.2	42.0 ± 3.5	–1.945	0.054

### Analysis of inflammatory factors

There was no significant difference in IL-6 levels between the sarcopenia and non-sarcopenia groups (*P* = 0.983); however, the levels of IL-10, IL-4, and TNF-α in the overall sarcopenia group were significantly higher than those in the non-sarcopenia group (*P* < 0.001, *P* = 0.001, and *P* < 0.001, respectively).

Regarding Han ethnicity, there was no significant difference in the level of IL-6 between the control group and the case group (*P* = 0.806). The levels of IL-10, IL-4, and TNF-α in the case group were higher than those in the control group (*P* = 0.006, *P* = 0.016, and *P* < 0.001, respectively). Regarding Uygur subjects, there was no significant difference in the level of IL-6 between the control group and the case group (*P* = 0.807), while the levels of IL-10, IL-4, and TNF-α in the case group were significantly higher than those in the control group (*P* = 0.001, *P* = 0.013, and *P* < 0.001, respectively) (Table 2).

**TABLE 2:** Inflammatory cytokines levels in difference nationality between sarcopenia and non-sarcopenia group.

Variables	Overall			Han nationality			Uygur nationality		
	Sarcopenia	Non-sarcopenia	*P*	Sarcopenia	Non-sarcopenia	*P*	Sarcopenia	Non-sarcopenia	*P*
IL-4 (pg/ml)	2.63 ± 0.66	2.27 ± 0.56	0.001	2.69 ± 0.74	2.34 ± 0.48	0.016	2.56 ± 0.57	2.21 ± 0.63	0.013
IL-6 (pg/ml)	1.85 ± 0.60	1.84 ± 0.53	0.983	1.89 ± 0.59	1.85 ± 0.62	0.806	1.81 ± 0.62	1.84 ± 0.44	0.807
IL-10 (pg/ml)	3.43 ± 0.92	2.72 ± 1.02	<0.001	3.27 ± 0.82	2.67 ± 1.03	0.006	3.59 ± 1.00	2.77 ± 1.03	0.001
TNF-α (pg/ml)	14.83 ± 3.40	6.76 ± 2.93	<0.001	14.12 ± 3.66	5.35 ± 2.91	<0.001	15.53 ± 3.00	8.16 ± 2.19	<0.001

After adjusting for sex, age, hypertension, blood lipid concentration, and obesity, the analysis of covariance revealed that the levels of IL-10, IL-4, and TNF-α in the case group were significantly higher than those in the control group (*P* < 0.001, *P* = 0.011, and *P* < 0.001, respectively) (Table 3).

**TABLE 3:** Analysis of inflammatory factors after adjusting for sex, age, hypertension, blood lipid concentration, and obesity.

Inflammatory cytokines	Groups	Mean	*F*	95% CI	*P*
IL-4	Non-sarcopenia	2.32	6.572	2.18–2.46	0.011
	Sarcopenia	2.59		2.45–2.73	
IL-6	Non-sarcopenia	1.87	0.225	1.73–2.00	0.636
	Sarcopenia	1.82		1.68–1.95	
IL-10	Non-sarcopenia	2.73	14.464	2.50–2.97	<0.001
	Sarcopenia	3.42		3.18–3.66	
TNF-α	Non-sarcopenia	7.16	160.948	6.40–7.91	<0.001
	Sarcopenia	14.41		13.66–15.16	

### Analysis of correlation between sarcopenia and clinical factors

Logistic analysis revealed that after adjusting for sex, age, hypertension, obesity, as well as IL-10, IL-4, and IL-6, an increased plasma level of TNF-α was significantly associated with sarcopenia (*P* < 0.001) (Table 4).

**TABLE 4:** Logistic analysis after adjusting for sex, age, hypertension, obesity, and interleukin (IL)-10, IL-4, and IL-6 levels.

	B	SE	*OR*	95% CI	*P*
Age	0.105	0.098	1.111	0.91–1.35	0.283
Gender	0.154	0.768	1.166	0.26–5.26	0.841
Hypertension	0.398	0.824	1.488	0.30–7.48	0.629
Triglyceride	0.206	0.503	1.228	0.46–3.29	0.682
Abdominal obesity	1.292	0.955	3.641	0.56–3.69	0.176
IL-4	1.140	0.630	3.127	0.91–10.75	0.070
IL-6	–0.304	0.704	0.738	0.19–2.94	0.667
IL-10	0.554	0.420	1.741	0.76–3.96	0.187
TNF-α	0.981	0.203	2.667	1.79–3.97	< 0.001

### Partial least squares-discriminant analysis model of serum inflammatory cytokines for sarcopenia

**FIGURE 2:**
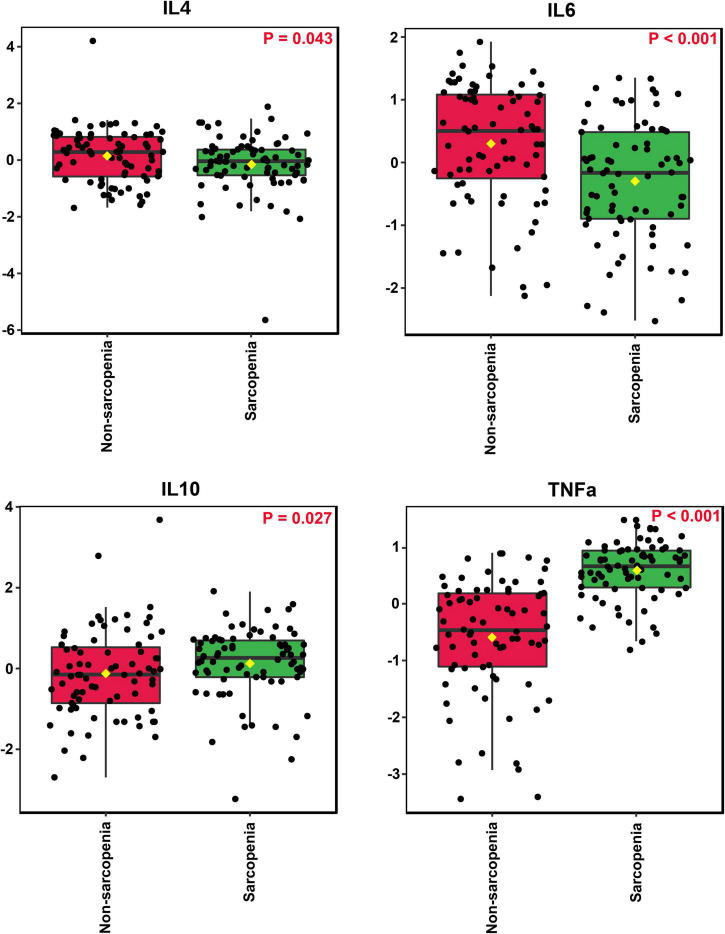
Comparison of the inflammatory cytokines of all individuals with and without sarcopenia.

**FIGURE 3:**
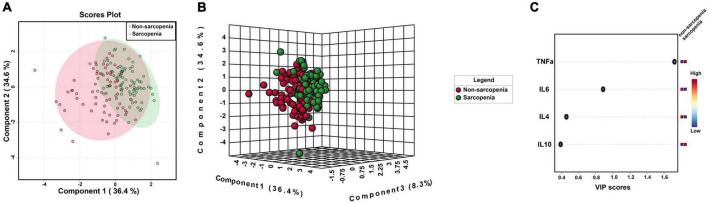
PLS-DA model of serum inflammatory cytokines for sarcopenia. **(A)** 2D clustering of sarcopenia vs. non-sarcopenia groups (*n* = 76 and *n* = 76, respectively) on PLS-DA; **(B)** 3D clustering of sarcopenia vs. non-sarcopenia groups (*n* = 76 and *n* = 76, respectively) on PLS-DA; **(C)** VIP scores of inflammatory cytokines between sarcopenia vs. non-sarcopenia groups.

**FIGURE 4:**
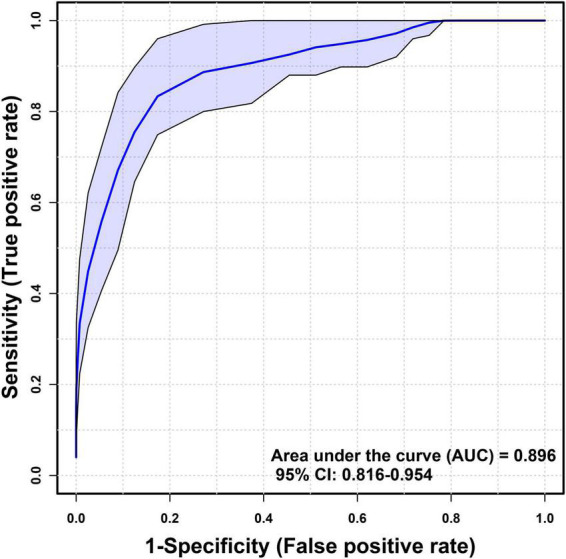
The area under the receiver operating characteristic curve performance for sarcopenia.

Inflammatory cytokines profiles of all individuals with and without sarcopenia were compared using MetaboAnalyst. The results indicated that four normalized inflammatory cytokines levels differed significantly between the two groups using Wilcoxon rank-sum testing (Figure 2). In addition, the 2D and 3D score plots from the PLS-DA models for inflammatory cytokines showed clear and robust separation between sarcopenia and non- sarcopenia individuals (Q2 value: 0.498, R2Y value: 0.526, confirming that they had acceptable validity), suggesting a strong correlation between sarcopenia and inflammatory cytokines (Figures 3A,B). The VIP results indicated that TNF-α contributed the most to sarcopenia classification (Figure 3C). Two inflammatory cytokines, including TNF-α and IL-4, were selected by PLS-DA to construct a model, which had an overall AUROC of 0.896 (95%CI 0.816–0.954) for sarcopenia (Figure 4).

## Discussion

We found that the plasma levels of IL-10, IL-4, and TNF-α in the case group were higher than in the control group. After adjusting for sex, age, hypertension, blood lipid concentration, and obesity, the level of TNF-α in the case group was higher than that in the control group, following that of IL-10 and IL-4, which indicated that an increased plasma level of TNF-α was significantly associated with sarcopenia.

Recently, exploring the potential role of inflammatory cytokines in sarcopenia has been a topic of great interest. A study by Rong et al. found that the elderly population with sarcopenia in China was correlated with increased levels of inflammatory cytokine IL-10, IL-6, and IL-6/IL-10 ratios (16). Moreover, a cross-sectional study (17) indicated that higher plasma levels of TNF-α were significantly associated with a 7.6-fold increased risk of sarcopenia. However, levels of soluble receptors of tumor necrosis factor-alpha were lower for sarcopenic community-dwelling elderly women residents of Brazil (18). Taken together, these findings suggest a role of high-grade inflammation in developing sarcopenia.

Inflammatory cytokines are a type of “information material” produced by white blood cells and other cells (e.g., nerve cells and glial cells) in the body. An inflammatory cytokine is a protein or small molecular polypeptide with an immune regulation function. Inflammatory factors act on target cells via autocrine and paracrine pathways, causing extensive biological effects (19). Inflammatory cytokines can be divided into pro-inflammatory and anti-inflammatory factors. With an increase in age, cytokine imbalances in the elderly manifest as an increase in pro-inflammatory factors, a decrease in anti-inflammatory factors, and finally lead to a chronic low-level inflammatory state, also known as “immune aging” (20). Interleukins are multifunctional cytokines produced by many cells *in vivo*, such as inflammatory cells, vascular endothelial cells, fibroblasts, adipocytes, and muscle cells. IL-4, IL-10, and IL-6 are anti-inflammatory factors (21). The influence of inflammatory cytokines on the elderly is multifaceted and includes aspects such as bone quality, muscle metabolism, and nutrition balance. In addition, proinflammatory factors can also increase platelet brittleness and affect coagulation pathways by affecting the endothelium. All of these directly or indirectly lead to an increased risk for cardiovascular events in the elderly (22). Various cytokines involved in the pathogenesis of sarcopenia have been identified in foreign studies and include TNF-α, IL-1, IL-2, IL-4, IL-6, IL-8, and IL-10.

Wang et al. (23) found that the expression of the pro-inflammatory factors MCP-1, IL-8, and IL-6 increased significantly in young and older men after strenuous exercise, while the expression of the anti-inflammatory factors IL-4, IL-10, and IL-13 increased only slightly. Della Gatta et al. (24) found that elderly individuals with elevated serum IL-6 levels (> 5 pg/ml) were more likely to experience decreased muscle mass and muscle strength. They confirmed that the increase in the serum soluble IL-6 receptor was closely related to a decrease in muscle mass. However, in a study involving 4,252 elderly men aged 60–79 years, Conte et al. (25) came to a different conclusion: Their results revealed no correlation between IL-6 and arm muscle circumference and non-fat BMI. In the present study, there was no significant difference in plasma IL-6 concentration between the sarcopenia and control group, which may be related to the complex mechanism(s) of IL-6 and may be explained by the different populations investigated.

TNF-α is mainly produced by activated macrophages, natural killer cells, and T lymphocytes. It is vital in malignant consumptive disease, also known as “cachexia.” As a typical proinflammatory cytokine, TNF-α is associated with many chronic diseases such as wasting syndrome, chronic infection, and metabolic disorder syndrome. However, the mechanism of TNF-α in the pathogenesis of sarcopenia remains unclear (26).

Lin and Yue (27) studied changes in muscle mass and muscle strength in 2,177 elderly patients during a 5-year follow-up. The authors found that TNF-α was negatively correlated with the cross-sectional area of the thigh muscle and with grip strength, suggesting that TNF-α negatively influenced muscle mass and grip strength in the elderly. A 4-year observation of individuals ≥ 85 years of age and 5-year observations of 70–79-year-old individuals demonstrated that plasma TNF-α levels were a predictor of a significant decrease in muscle strength. Grip strength decreased by 1.2–1.3 kg for each standard deviation increase in the TNF-α level (28). Similarly, Schaap et al. (29) found that mice with TNF-α gene transposition produced a large amount of TNF-α and exhibited thigh muscle fiber atrophy and growth restriction. The present study demonstrated that the concentration of TNF-α in the sarcopenia group was significantly higher than in the control group. Logistic analysis revealed that after adjusting for sex, age, hypertension, obesity, IL-10, IL-4, and IL-6, an increased plasma level of TNF-α was significantly associated with sarcopenia, which was consistent with the results of previous studies, suggesting that TNF-α may play an essential role in the pathogenesis of sarcopenia.

IL-10 is an anti-inflammatory cytokine derived from T-helper 2 and other cells. It acts on macrophages, down-regulates the expression of major histocompatibility complex II, weakens anti-inflammatory presentation, and reduces inflammatory reaction(s) (30). Coletti et al. (30) found no significant difference in the concentration of IL-10 between patients with sarcopenia and the control group. Another study reported that the number of anti-inflammatory cytokines decreased with increases in adipose tissue (31). The present study investigated the relationship between the plasma pro-inflammatory factors TNF-α and IL-6 and the anti-inflammatory factors IL-4 and IL-10 in the elderly population and sarcopenia. The results showed that increases in the concentration of the plasma pro-inflammatory factor TNF-α and the anti-inflammatory factors IL-4 and IL-10 were related to sarcopenia, which was contradictory to the definition of “immune aging.” This may be explained by the chronically low level of inflammation in patients with sarcopenia. In response to stimulation of the proinflammatory factor TNF-α, the body secretes IL-4 and IL-10 to participate in the anti-inflammatory process. After further correction of plasma IL-10 and IL-4 levels, increased plasma TNF-α level remains associated with sarcopenia, suggesting that immune aging may be involved in the pathogenesis of sarcopenia.

Dysregulation of the cytokine network is deemed the major element speeding up the aging process and causing related illnesses (32). Cytokine interaction constitutes a cytokine network. It can be divided into two types: Pro-inflammatory cytokine network and anti-inflammatory cytokine network (33). Changes in inflammatory cytokine networks control the direction of inflammation. One of the main characteristics of aging is the chronic progressive increase of inflammatory response, which is called inflammatory aging and is closely related to immune aging. Increasing the level of serum inflammatory cytokines such as interleukin-6 (IL-6) and tumor necrosis factor-α (TNF-α) in the elderly are considered risk factors for cardiovascular and degenerative diseases (34). Our study found that the tumor necrosis factor α level in patients with sarcopenia was higher than that of patients without sarcopenia, which concludes that inflammatory factor network disorder is associated with sarcopenia. Inflammatory cytokines network disorder is mainly a response to tissue damage, which results from the age-related reduction in hemoglobin levels and consequent lower tissue oxygenation (35). Thus, IGF, growth hormone, testosterone, and nutritional supplements may be plausible strategies to improve inflammation and increase muscle mass. In addition, the exercise effect on anabolic hormone production has been well studied. Therefore, the optimal combination to treat sarcopenia must include personalized multicomponent exercise and supplementation with proteins and micronutrients to prevent deficiencies in the elderly (36).

A major limitation of our study is a relatively small sample size, which leads to our findings remaining exploratory. However, we have clearly shown an increased plasma level of TNF-α in individuals with sarcopenia with a clear separation compared to a healthy control using PLS-DA plus logistic regression analysis. In addition, using a cross-sectional rather than a longitudinal survey prevented us from disease progression and dietary consideration. Neither the amount of physical activity nor nutritional patterns were quantified in the present study. The study’s strengths provide initial information on a unique inflammation profile in the elderly natural population with sarcopenia in a cross-sectional study in the agricultural and pastoral areas of Xinjiang of China. A large-scale, multicenter study will be indispensable for validating these findings and determining the role of inflammatory cytokines and whether the profile is altered before sarcopenia develops.

## Conclusion

This study confirmed that an increased plasma level of TNF-α was associated with sarcopenia in elderly individuals residing in the agricultural and pastoral areas of Xinjiang, China. In the future, we intend to perform a cohort study to explore the causal relationship(s) between serum inflammatory markers and sarcopenia and the mechanism of sarcopenia development.

## Data availability statement

The original contributions presented in this study are included in the article/supplementary material, further inquiries can be directed to the corresponding author/s.

## Ethics statement

The studies involving human participants were reviewed and approved by the Ethics Committee of People’s Hospital of Xinjiang Uygur Autonomous Region. The patients/participants provided their written informed consent to participate in this study.

## Author contributions

HW contributed to the study design, data collection and analysis, results interpretation, and provided critical manuscript revisions. AW and ZM wrote the article. WX, TM, JL, and SX involved in the data analysis and interpretation and also provided critical manuscript revisions. All authors read and approved the submitted manuscript.

## Footnotes

1
https://www.metaboanalyst.ca
